# Herbal Medicines and Drugs Interactions: Cytochrome P450 Responsibility

**DOI:** 10.2174/0109298673428595251209114801

**Published:** 2026-02-26

**Authors:** Mariia Shanaida, Oleksandra Oleshchuk, Oksana Shevchuk, Kateryna Posokhova, Yana Ivankiv, Khrystyna Mocherniuk, Mykola Klantsa, Mykhaylo Korda

**Affiliations:** 1 Department of Pharmacognosy and Medical Botany, I. Horbachevsky Ternopil National Medical University, Ternopil, Ukraine;; 2 Department of Pharmacology and Clinical Pharmacology, I. Horbachevsky Ternopil National Medical University, Ternopil, Ukraine;; 3 Department of Medical Biochemistry, I. Horbachevsky Ternopil National Medical University, Ternopil, Ukraine

**Keywords:** Herb-drug interaction, medicinal plants, phytoconstituents, cytochrome P450, CYP450 inducers, CYP450 inhibitors, pharmacovigilance

## Abstract

Despite the global prevalence of herbal medicine use and the common perception of their safety, substantial evidence indicates a significant potential for clinically relevant herb-drug interactions that may affect the pharmacokinetics and pharmacodynamics of co-administered pharmaceuticals. This critical issue poses a considerable threat to patient safety and the effectiveness of conventional treatments, primarily through modulation of the cytochrome P450 (CYP450) enzyme system, a key metabolic pathway for many drugs. This research aims to elucidate how herbal remedies affect CYP450 enzyme activity, thereby influencing drug pharmacokinetics. Our methodology involves a comprehensive critical evaluation of existing scientific data from *in silico*, *in vitro*, and *in vivo* studies, clinical trials, and meta-analyses to identify specific herbal medicines with significant effects on CYP450. The investigation outlines their mechanisms of action, distinguishing between enzyme induction, which can diminish drug efficacy, and inhibition, which may lead to increased drug concentrations and toxicity. It was highlighted that certain medicinal plants and their bioactive compounds may act as inducers or inhibitors across major isoforms, including CYP1A2, CYP2C9, CYP2D6, and CYP3A4. Comprehensive data were compiled for ten plant species with the most extensive scientific information regarding their effects on CYP450, namely St John’s wort (*Hypericum perforatum* L.), Echinacea (*Echinacea purpurea* (L.) Moench), Ginkgo (*Ginkgo biloba* L.), Danshen (*Salvia miltiorrhiza* Bunge), Garlic (*Allium sativum* L.), Ginger (*Zingiber officinale* Roscoe), Milk thistle (*Silybum marianum* L. Gaertn.), Black cohosh (*Actaea racemosa* L.), Ginseng (*Panax ginseng* C.A. Mey), and Liquorice (*Glycyrrhiza glabra* L.). Ultimately, this study underscores the vital role of the CYP450 system in mediating these interactions and advocates for increased awareness among healthcare professionals and patients. Looking ahead, conducting robust, standardised clinical trials, developing predictive interaction models, and performing comprehensive analyses of herbal constituents are crucial to ensuring safe and effective pharmacotherapy involving herbal medicines.

## 1 INTRODUCTION

The global landscape of healthcare has witnessed a significant surge in the use of herbal medicines over the past few decades, with millions of individuals worldwide incorporating them into their therapeutic regimens for both curative and prophylactic purposes. This widespread acceptance is often underpinned by a perception of inherent safety and efficacy, stemming from their ‘natural’ origin. Many consumers take herbal remedies alongside conventional medications without informing their healthcare providers. Unfortunately, such combinations can lead to adverse side effects, particularly when herbal products interact with drug-metabolising enzymes and transporters [1]. Evaluating the potential for interactions between herbal products and medications is complicated by variations in the composition of these products, uncertainties regarding their active ingredients, and limited knowledge about the pharmacokinetics of these compounds.

The rise in the use of both conventional medications and herbal supplements has made herb-drug interactions a significant concern in clinical practice [2]. Unlike drug-drug interactions, which have been extensively researched, herb-drug interactions are less well understood due to the complexity and often poorly characterised nature of various herbal components. Nevertheless, such interactions can lead to reduced therapeutic efficacy, adverse effects, or even toxicity, highlighting the need for effective prediction and management [2].

Generally, medicinal plants are an important source of pharmaceuticals. They are traditionally used by millions of people for medicine, food, and drink in both developed and developing countries. Over the last few decades, a marked increase has been observed in the pharmaceutical market for herbal medicines. Its market size was valued at USD 233.08 billion in 2024 and, according to Fortune Business Market, is expected to grow at a CAGR of 8.23% during 2025-2032 [3]. However, the interaction of herbal medicines with other drugs may cause serious adverse effects or reduce their efficacy [4, 5]. Herbal products, being heterogeneous mixtures of numerous bioactive compounds, possess substantial potential for clinically relevant drug interactions, particularly those mediated through the cytochrome P450 (CYP450) enzyme system [6, 7]. The interactions between popular herbal medicines and conventional drugs, including St John’s wort, ginkgo, ginseng, echinacea, garlic, and others, have been thoroughly analysed through numerous case reports and clinical trials [8].

CYP450 enzymes represent a superfamily of monooxygenases pivotal to the metabolism of a vast array of endogenous and exogenous compounds, including a significant proportion of currently prescribed pharmaceutical drugs. Their activity profoundly influences the pharmacokinetics of these medications, thereby dictating their therapeutic efficacy and safety profiles [9]. The diverse phytochemicals present in herbal medicines can critically modulate the activity of these enzymes, leading to intricate pharmacokinetic interactions. Such modulation, whether through enzyme induction (increased activity) or inhibition (decreased activity), can result in altered drug concentrations within the body, potentially causing subtherapeutic levels and treatment failure, or conversely, supratherapeutic levels and an increased risk of adverse drug reactions [10, 11].

The most well-known example of herbal medicine- drug interactions involving altered CYP450 activity, which pharmacologically reduces the effectiveness of a wide range of medications, is St John’s wort (*Hypericum perforatum* L.) [12]. Beyond the influence of specific herbal compounds, the inherent variability in CYP450 enzyme expression among individuals-shaped by genetic polymorphisms, environmental exposures, and dietary habits-further complicates the predictability and clinical management of herb-drug interactions [13]. Given these complexities, it is practically important for healthcare providers to have a comprehensive understanding of the potential for such interactions and the underlying biochemical mechanisms that govern these effects.

This review aims to systematically elucidate the intricate relationship between commonly used herbal medicines and drug interactions primarily mediated by the CYP450 enzyme system. By highlighting specific medicinal plants with well-characterised CYP450-modulatory activities, we intend to provide a comprehensive, updated overview that will serve as a valuable resource for clinicians, pharmacists, and researchers in navigating the implications of integrating herbal medicine use with conventional pharmacotherapy. Our methodology involved a critical evaluation of existing scientific data from *in silico*, *in vitro*, and *in vivo* studies, clinical trials, and meta-analyses to identify specific herbal medicines with significant effects on CYP450. The study outlines their mechanisms of action, distinguishing between enzyme induction, which can diminish drug efficacy, and inhibition, which may lead to increased drug concentrations and toxicity.

The literature search utilised the Scopus, Web of Science, PubMed, and ClinicalTrials.gov databases. The selection criteria comprised peer-reviewed articles, clinical studies, case reports, and relevant book chapters published primarily within the last two decades (2005-2025). Evidence was gathered from *in silico* predictions, *in vitro* experiments, *in vivo* animal models, clinical trials, and meta-analyses. To ensure clinical relevance, we focused on plant species with the most consistent and reproducible findings related to the modulation (induction or inhibition) of major CYP450 isoforms.

## 2 COMPLETED CLINICAL TRIALS REGARDING HERB-DRUG INTERACTIONS

When searching for clinical trials related to drug-herb interactions, we applied filters using keywords such as “herb-drug interactions” and the Latin names of specific plants. We also included a filter for “completed trials.” After conducting our search, we identified 17 completed clinical trials. However, we excluded some of these trials because they focused on complex, multi-component prescriptions of traditional Chinese medicine rather than on specific plant species. Some examples of completed clinical trials that examine herb-drug interactions are provided in Table **1** [14-22]. The studies presented in Table **1** were conducted in different countries, which indicates a significant interest of the scientific community and clinicians in this issue.

The research related to clinical trial NCT00029263 [23], concluded that data from conventional buffer-based *in vitro* studies were less predictive of botanical-drug interactions than *ex vivo* assessments. To validate this methodology, researchers utilised human plasma and serum samples collected from healthy subjects who had been administered either milk thistle or goldenseal extracts. These samples were used in incubation studies to assess their potential inhibitory effects on the enzymes CYP2C9 and CYP3A4/5, respectively. The study focused on silybin A and B, two primary phytochemicals in *Silybum marianum*, as well as hydrastine and berberine, which are considered the active constituents of *Hydrastis canadensis*. Both phosphate buffer and human plasma-based *in vitro* incubation systems were employed for evaluation. The researchers proposed that this novel *ex vivo* approach may offer a more effective means of predicting clinically relevant botanical-drug interactions compared to conventional *in vitro* methods.

In relation to the other completed clinical trials listed in Table **1**, no published papers reporting their findings are currently available. An analysis of Table **1** reveals that these clinical trials focused on studying the effects of plant extracts derived from *Echinacea purpurea* [17, 19, 24], *Hypericum perforatum* [15], and *Hydrastis canadensis* [14]. There have also been studies investigating the effects of individual compounds extracted from medicinal plants, including the combined use of green tea extract and soy isoflavones [22], as well as cannabidiol [21] and resveratrol [20]. Most of the identified studies regarding herb-drug interactions were interventional, with the exception of two, which were observational [24, 25]. Studies on the drumstick tree (*Moringa oleifera*) have reported CYP450-modulating effects. Phytochemical investigations of *Moringa oleifera* leaves identified polyphenols and terpenoids that inhibit CYP3A4 *in vitro* [26].

## 3 CYTOCHROME P450 DRUG INTERACTIONS - GENERAL CONSIDERATIONS

The main effects of drugs on the human body are studied by pharmacodynamics, while the body’s influence on drugs is the domain of pharmacokinetics. The abbreviation ADME describes the key processes involved in drug transformation in the body: A - Absorption, D - Distribution, M - Metabolism, and E - Elimination [27]. The liver, gut, and kidneys are the primary organs responsible for these processes [9, 27].

Historically, metabolism or biotransformation was typically described as consisting of only two phases-Phase I and Phase II-usually occurring in sequence. However, some drugs can undergo Phase II biotransformation directly, without prior Phase I reactions. Excretion was traditionally considered a separate process. Today, a three-phase model is increasingly recognized:

Phase I - Modification: Involving oxidation and reduction reactions, this phase introduces or exposes functional groups in xenobiotics.

Phase II - Conjugation: Endogenous molecules are attached to intermediate metabolites in this phase.

Phase III - Excretion: This phase involves the elimination of xenobiotics or their metabolites from the cell.

This paper focuses on metabolism, particularly Phase I modification by CYP450 enzymes, due to their pivotal role in herbal-drug interactions. This discovery was significant in biochemistry and pharmacology, as it linked P450 enzymes to crucial biological processes, including hormone synthesis, drug metabolism, and detoxification. Diverse factors-including sex, age, nutrition, concomitant diseases and medications (drug-drug interactions when multiple drugs are used simultaneously), genetic profile, and others-impact biotransformation processes [28, 29]. CYP450s are also responsible for first-pass metabolism [30], a presystemic phenomenon that reduces the concentration of active drugs before they reach the site of action or systemic circulation.

Today, CYP450 is recognized as a superfamily with five main classes based on redox partners and interaction relationships [31, 32]. However, most researchers classify CYP450 enzymes based on amino acid sequence homology [13]. Three major families of cytochrome P450 enzymes have been extensively investigated and are identified by Arabic numerals (*e.g.*, CYP1 or CYP2), followed by a subfamily letter (*e.g.*, CYP1A or CYP2C), and individual enzymes or isoforms are designated by specific numbers (*e.g.*, CYP1A2 or CYP2C19) [33]. For example, CYP2D6 refers to Cytochrome P450 family 2, subfamily D, member 6, while CYP3A4 refers to Cytochrome P450 family 3, subfamily A, member 4. It is well established that CYP1A2, CYP2C9, CYP2C19, CYP2D6, CYP2E1, and CYP3A4 are the most active enzymes in hepatic xenobiotic metabolism, including various medications. Other CYP450 types also play important roles in numerous endogenous functions.

CYP450 is named for its characteristic maximum absorption at 450 nm in the reduced state in the presence of carbon monoxide [13]. CYPs catalyse monooxygenase reactions, particularly involving steroid compounds [34], which is why the term “monooxygenase system” is often used in the literature. They are highly versatile biocatalysts, participating in over 20 different types of chemical oxidation reactions, including hydroxylation, epoxidation, decarboxylation, N- and O-dealkylation, nitration, and C-C bond coupling or cleavage.

Regulatory agencies such as the U.S. Food and Drug Administration (FDA) and European Medicines Agency (EMA) require preclinical and clinical studies to include information on key CYP450 isoforms- CYP1A2, CYP2B6, CYP2C8, CYP2C9, CYP2C19, CYP2D6, and CYP3A-because most clinically used drugs undergo biotransformation *via* these enzymes (FDA, 2020; FDA, 2017; EMA, 2012) [35]. Each family of CYP1, CYP2, and CYP3, which are extensively involved in drug and herbal metabolism, possesses unique characteristics relevant to pharmacokinetics and herb-drug interactions.

Central mechanisms resulting in clinically significant drug-drug and herbal-drug interactions involve the inhibition and induction of CYP450 enzymes. Numerous substances can modify the activity of these enzymes. CYP450 inducers increase enzyme expression and activity, leading to faster drug metabolism and reduced drug concentrations in the body. Conversely, CYP450 inhibitors decrease or block enzyme activity, resulting in slower metabolism and increased drug concentrations [27, 36, 37]. Furanocoumarins are the primary compounds responsible for the food-drug interactions known as the grapefruit effect, caused by inhibition of CYP3A4-mediated drug metabolism. CYP450 inhibition can be reversible-either competitive or non- competitive-or irreversible/quasi-irreversible (mechanism-based inhibition) [36].

The most common and well-known inducers of CYP450 include rifampin, barbiturates, carbamazepine, chronic alcohol consumption, and St John’s wort (*Hypericum perforatum*). Well-known inhibitors include amiodarone, cimetidine, omeprazole, ritonavir, ketoconazole, chloramphenicol, grapefruit juice, and others.

The primary mechanisms for CYP3A4 induction involve increased synthesis of the enzyme *via* upregulation of gene expression, stabilization of mRNA molecules, and a decreased rate of enzyme catabolism [38].

## 4 CYP-MEDIATED HERB-DRUG INTERACTIONS

**Fig. (1):**
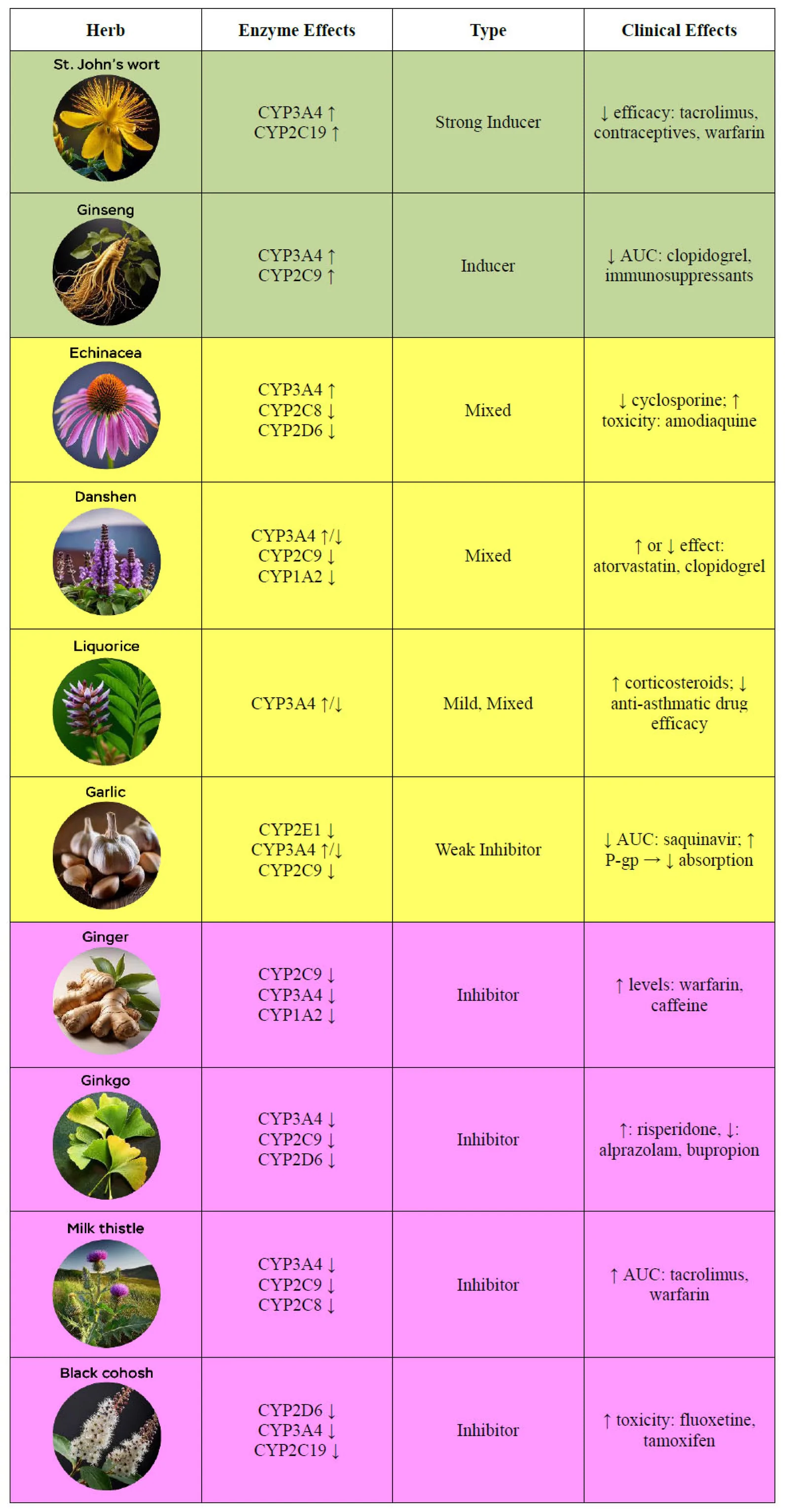
The primary species of medicinal plants for which a significant number of scientific publications were identified regarding their effect on CYP450 enzymes.

Unlike conventional medications, herbal products comprise complex blends of biologically active compounds, making the likelihood of herb-drug interactions higher than that of drug-drug interactions [39-41]. Ten species of medicinal plants were identified for which the maximum amount of scientific data regarding their impact on CYP450 is available (Fig. **1**). These plant species were selected based on the availability of strong scientific evidence demonstrating their effects on cytochrome P450 enzymes, specifically CYP1A2, CYP2C9, CYP2D6, and CYP3A4. Only species with reproducible data from preclinical and clinical studies were included to ensure a focus on clinically relevant herb-drug interactions.

### 4.1 St John’s Wort (*Hypericum perforatum*)

St John’s wort (*Hypericum perforatum* L., Hypericaceae family) is a perennial herbaceous plant. Recently, it has been considered one of the main medicinal crops in Europe [42]. The chemical composition of this plant has been extensively studied, and its well-documented pharmacological effects include antibacterial, antiviral, anti-inflammatory, and antidepressant properties [43, 44]. Many of these pharmacological effects are attributed to hypericin, a naphthodianthrone, as well as various flavonoid constituents. Hypericin is also known to cause photosensitive reactions associated with St John’s wort. Recent research has shown that hyperforin, a prenylated phloroglucinol, is the primary compound responsible for its antidepressant effects [43]. Today, hyperforin is also considered a potent anticancer agent and a valuable compound for treating Alzheimer’s disease [42].

St John’s wort is recognized as a strong inducer of multiple CYP450 enzymes, primarily due to hyperforin. The FDA classifies St John’s wort as a potent inducer of both CYP450 isoenzymes and the drug transporter P-glycoprotein, potentially lowering the area under the curve (AUC) of co-administered drugs by up to 80% [45-49]. The degree of enzyme induction depends significantly on the hyperforin concentration in a given preparation [50, 51]. Notably, CYP3A4, CYP2E1, and CYP2C19 are markedly upregulated, whereas the effects on CYP1A2, CYP2C9, and CYP2D6 are minimal [52]. The underlying mechanism involves St John’s wort binding to the pregnane X receptor (PXR). Once activated, PXR translocates to the nucleus and binds with retinoid X receptors to form a functional complex, which then binds DNA response elements, promoting transcription and increasing expression of enzymes such as CYP3A4 [53-55].

Short-term administration of St John’s wort (1-3 days) does not significantly alter enzyme levels, but regular use over 14 days is sufficient to establish a stable state of induction [52, 56]. Despite this, St John’s wort is less potent than other known inducers, such as rifampin, carbamazepine, and bosentan; when combined with these agents, overall enzyme activity is determined by the stronger inducer [57]. The extent of induction also varies according to the St John’s wort product, dosage, and hyperforin content [50]. EMA guidelines recommend a daily dose of 500 mg of *Hypericum perforatum* dry extract, standardized to approximately 0.96 mg of hyperforin [58].

High-hyperforin formulations of St John’s wort can reduce plasma concentrations of CYP-metabolized drugs by accelerating their clearance, occasionally resulting in therapeutic failure [50, 56, 59, 60]. In contrast, low-hyperforin formulations (≤0.2%) generally do not produce significant interactions with major CYP isoforms or P-gp, as demonstrated in studies involving drugs such as caffeine, bupropion, flurbiprofen, omeprazole, dextromethorphan, midazolam, and fexofenadine [61]. A study in 11 healthy subjects evaluated the effects of *Hypericum perforatum* on CYP3A4 by administering 300, 900, and 1800 mg daily for 14 days. Using microdoses of midazolam, an FDA-recommended CYP3A probe, researchers observed 1.96-, 3.86-, and 5.62-fold increases in oral clearance, respectively [35, 57]. CYP450 activity impacts the metabolism of oral contraceptives, bupropion, digoxin, fexofenadine, midazolam, imatinib, rifampicin, rivaroxaban, verapamil, simvastatin, warfarin, and other drugs [47, 48, 52, 54, 62-72].

Bioactive compounds in St John’s wort, including hyperforin, flavonoids, biflavones, acylphloroglucinols, and phenylpropanes, may also alter warfarin metabolism *via* CYP3A4 and CYP2C9, increasing the risk of reduced anticoagulation or bleeding [73]. Recent scientific studies have provided thorough assessments of databases and references concerning herb– drug interactions and related adverse outcomes [12, 74-77]. According to the Mayo Clinic, the use of St John’s wort in combination with drugs metabolized by CYP1A2, CYP2B6, CYP2C19, CYP2C9, and CYP3A4 is discouraged due to the risk of significant interactions [78]. Conversely, St John’s wort may potentiate the therapeutic effects of prodrugs, such as clopidogrel, that rely on CYP enzyme-mediated bioactivation [79].

Genetic variability influences baseline liver CYP450 activity, affecting drug metabolism [80, 81]. Studies suggest that individuals with ultra-rapid or extensive metabolic capacity experience more pronounced interactions when taking *Hypericum perforatum* [56].

In a randomized crossover study, 18 healthy volunteers received 5 mg doses of either immediate- or prolonged-release tacrolimus, both alone and following *Hypericum perforatum* administration. It reduced the AUC and Cmax of immediate-release tacrolimus to 73% and 61%, respectively, and of prolonged-release tacrolimus to 67% and 69%. Notably, those with higher baseline CYP3A4 activity or expressing CYP3A5 exhibited less pronounced effects [69]. In an animal study, male mice received St. John's wort extract at a dose of 0.6 g/kg/day for 21 days, leading to enhanced hepatic oxidation of nifedipine (a CYP3A substrate). After 28 days, the enzymatic activity for nifedipine oxidation and warfarin 7-hydroxylation (a CYP2C pathway) rose by 95% and 34%, respectively. Immunoblotting showed increased CYP2C protein expression, while enzymes linked to CYP1A, CYP2A, CYP2B, CYP2D, and CYP2E1 remained unchanged [82].

The animal studies also confirm the induction of CYP3A isoforms in response to St. John's wort. While hyperforin activates the PXR, it does not fully account for changes observed in CYP3A23-3A1 expression and activity. The data suggest additional, unidentified constituents in *Hypericum perforatum* may also modulate CYP3A *in vivo* and *in vitro*, paralleling findings in humans [83].

### 4.2 Echinacea (*Echinacea purpurea*)

Echinacea (*Echinacea purpurea* (L.) Moench) is a perennial plant belonging to the Asteraceae family and is native to eastern North America [84]. The primary components found in the roots, rhizomes, and above- ground parts include alkylamides, glycoproteins, polysaccharides, flavonoids, and phenolic acids, mainly caffeic acid derivatives. Various Echinacea species, including *E. purpurea*, *E. angustifolia* DC, and *E. pallida* Nutt., exhibit numerous beneficial biological effects, such as immunostimulation, anti-inflammatory properties, and antimicrobial activity [85, 86]. However, phytochemical and pharmacological properties can vary between extracts from different sources.

*E. purpurea* can induce the expression of CYP1A2, CYP3A4, and the P-glycoprotein transporter *via* the PXR signaling pathway in HepG2 cell cultures [87]. PXR is a key transcription factor regulating the expression of enzymes and transporters involved in xenobiotic metabolism, including Phase I and Phase II drug biotransformation. PXR activation is considered a central mechanism in many herb-drug interactions that alter pharmacokinetics and affect therapeutic outcomes [88].

Evidence supports a role for *E. purpurea* in upregulating CYP3A4 activity [89]. One study reported a significant increase in midazolam clearance, potentially due to CYP3A4 induction and/or activation of the organic anion transporter OATP1A2 [46]. While *E. purpurea* does not alter plasma levels of Paxlovid (ritonavir + nirmatrelvir) despite possible CYP3A induction, a reduction in plasma warfarin levels *via* CYP3A activation was observed [90]. Modulation of CYP3A4 by Echinacea may affect the efficacy of anticancer therapies, as highlighted in reviews addressing interactions between herbs, foods, dietary supplements, and 117 anticancer drugs, with particular focus on CYP3A4, CYP2D6, CYP1A2, and CYP2C8 [40, 91].

Although *E. purpurea* is identified as a weak CYP3A4 inducer, its effect on docetaxel pharmacokinetics is minimal. Conversely, a potentially significant interaction with cyclophosphamide (administered at 1600 mg daily) was reported, possibly *via* CYP3A4 inhibition [92]. Some studies show inconsistencies: *E. purpurea* reduced oral clearance of CYP1A2 substrates but had little effect on CYP2C9 or CYP2D6-metabolized drugs and differentially influenced CYP3A activity in liver *versus* intestinal tissues [64]. Other investigations report negligible effects on drug-metabolizing enzymes.

*In vitro* and animal studies indicate that standardized *Echinacea* extracts can suppress CYP3A mRNA levels and weakly inhibit CYP2C8 (IC_50_ > 10 µg/mL). Typical oral doses yield plasma concentrations far below those required for inhibition, suggesting a low risk of clinically relevant interactions [87, 88]. Consistent with these findings, Matura *et al.* [89] reported that *E. purpurea* did not significantly alter CYP3A4 or CYP2D6 gene expression, supporting minimal likelihood of herb-drug interactions at standard dosages.

### 4.3 Ginkgo (*Ginkgo biloba*)

Ginkgo (*Ginkgo biloba* L.) is a deciduous tree of the Gymnosperms, native to China and now thriving in temperate climates. Its leaves have been used in traditional Chinese medicine for centuries [93], and currently, *Ginkgo biloba* leaf extract is of significant interest for vascular protection [94]. The extract is known for cerebroprotective effects and has been used to address age-related memory deficits, including those associated with Alzheimer’s disease and dementia. Flavonoids, key medicinal components of *Ginkgo biloba*, inhibit xanthine oxidase and platelet-activating factor receptors, contributing to neuroprotective and antihypertensive effects [95]. Ginkgetin, a biflavonoid dimer isolated from Ginkgo, is recognized for its neuroprotective potential [96].

Over the past decade, bilobalide, a sesquiterpene trilactone from Ginkgo leaves, has been extensively studied for neuroprotective and cardiovascular effects, largely through antioxidant and anti-inflammatory mechanisms [94, 97]. Recent literature highlights Ginkgo’s impact on drug pharmacokinetics and pharmacodynamics, primarily *via* inhibition of CYP3A4, CYP2C9, CYP1A2, and CYP2E1 [54, 98-101].

CYP inhibition mechanisms are classified as reversible or irreversible [102]. Reversible inhibition involves direct competition between substrate drugs and inhibitors for enzyme binding sites and can be competitive, non-competitive, uncompetitive, or mixed, with competitive and non-competitive inhibition most commonly observed in herb-drug interactions.

Preclinical studies demonstrated that Ginkgo extract (100 mg/kg for 30 days) increased renal tissue concentrations of venlafaxine, while 200 mg/kg elevated fluoxetine levels in liver, kidney, and brain [103]. Daily administration of 360 mg Ginkgo extract for four weeks increased midazolam AUC by 25% [54]. Clinical reports associate co-administration of risperidone and Ginkgo with priapism, likely due to CYP2D6 and CYP3A4 inhibition [104].

Animal studies show Ginkgo increased both AUC and Cmax of metoprolol *via* CYP2D6 inhibition. Similarly, midazolam AUC and Cmax increased when co-administered with Ginkgo due to reduced CYP3A4 activity [104]. In rats, Ginkgo leaf tablets (ginkgolides A/B, bilobalide, quercetin, kaempferol) elevated amlodipine pharmacokinetic parameters. Co-incubation with ginkgolide B, bilobalide, or quercetin extended amlodipine’s half-life substantially [105].

Biflavones such as bilobetin, ginkgetin, isoginkgetin, and amentoflavone were identified as major CYP3A4 inhibitors, affecting drugs like tamoxifen, gefitinib, and ticagrelor [106]. An *in vitro* study using amodiaquine N-desethylation as a marker revealed moderate CYP2C8 inhibition through a mixed mechanism [107].

Combination of Ginkgo and sodium aescinate induced cefuroxime-related acute kidney injury in rats, likely due to competition for plasma protein binding and hepatic metabolism *via* CYP2C9 and CYP3A4 [108]. Conversely, Ginkgo has been reported to induce CYP enzymes or exert dual effects, decreasing concentrations of medications such as alprazolam, atorvastatin, simvastatin, metformin, tolbutamide, omeprazole, lopinavir, and nifedipine *via* interactions with CYP3A4, CYP2C9, and CYP2C19 [49, 84, 105-107].

In healthy volunteers, 240 mg/day Ginkgo extract for two weeks reduced alprazolam AUC by 17%, and CYP2C19 induction decreased omeprazole exposure in a genotype-dependent manner. However, other studies using CYP2C19 probe substrates did not confirm this effect, and current evidence for CYP3A4- or CYP2C9- mediated interactions remains inconsistent [49].

A systematic review [104] indicated that 14 days of Ginkgo administration had no major effect on bupropion AUC but shortened its half-life and increased the Cmax of its metabolite, reflecting CYP2B induction. Ginkgo intake has also been linked to decreased efficacy of trazodone due to CYP3A4 activation and breakthrough seizures in patients on antiepileptic drugs, with effects resolving upon discontinuation. Ginkgo reduced efavirenz AUC and Cmax but did not significantly affect bupropion pharmacokinetics at 120 mg twice daily for 14 days [104].

In rats, co-administration of Ginkgo with losartan increased its metabolism by 25%, likely due to CYP2C11 induction [109]. Single and repeated doses of Ginkgo tablets (100-200 mg/kg) with rosiglitazone showed dual modulation of CYP2C8, reducing metabolism after a single dose but enhancing it with repeated administration [110]. Hydrolyzed ginkgolides (0.1-10 μg/mL) in human liver microsomes and hepatocytes did not notably inhibit key CYP enzymes, but the highest concentration increased CYP3A4 activity and mRNA expression by 4.6-fold and 17.2-fold, respectively, indicating potential clinical relevance [111].

*In vitro* studies using rat liver microsomes showed Ginkgo extract enhanced clopidogrel conversion to its active metabolite. *In vivo*, high-dose Ginkgo reduced clopidogrel AUC and Cmax while increasing the same parameters for its active metabolite, demonstrating dose-dependent modulation of metabolism [112].

A clinical study of Ginkgo terpene lactone meglumine injection (containing ginkgolides A, B, and K) found no significant effect on midazolam metabolism or CYP3A4 activity after 8 and 14 days of administration in healthy volunteers [113]. Similarly, an 8-day treatment with Ginkgo extract (120 mg twice daily or 240 mg once daily) did not alter the *in vivo* activity of CYP1A2, CYP2C9, CYP2C19, or CYP2D6 in healthy volunteers [114]. Awortwe *et al.* documented several drug interactions involving *Ginkgo biloba*, including increased viral load when co-administered with efavirenz, zidovudine, and lamivudine, likely due to CYP3A4 induction. Seizure events were also reported, attributed to CYP2C19-mediated reductions in antiepileptic efficacy when Ginkgo was combined with valproic acid and phenytoin [73].

Critical analysis has shown substantial variability in *Ginkgo biloba*’s effects on CYP450 enzymes, particularly CYP3A4, CYP2C9, and CYP2D6. Some studies report clear inhibitory effects, others suggest enzyme induction, and some show no significant impact. This duality complicates interpretation and likely reflects methodological differences between studies. Several clinical trials reported a ~25% increase in midazolam AUC following Ginkgo administration, suggesting CYP3A4 inhibition [49, 95-98]. In contrast, other studies documented decreased alprazolam plasma concentrations after 2-4 weeks of standardized Ginkgo intake (240 mg/day), consistent with CYP3A4 induction [49, 84, 105-107]. Similarly, Ginkgo co-administration with risperidone was associated with priapism, likely due to CYP2D6 and CYP3A4 inhibition, whereas interactions with trazodone and several antiepileptic drugs suggested CYP induction [67, 101]. Animal studies further demonstrate dose-dependent inhibition of CYP2D6 (↑ metoprolol plasma levels) and induction of CYP2C11 and CYP3A1, affecting losartan and rosiglitazone metabolism [100, 101]. *In vitro* studies with isolated biflavones (amentoflavone, ginkgetin, isoginkgetin) show strong CYP3A4 inhibition, aligning with some-but not all-clinical findings [102].

Taken together, *Ginkgo biloba* exhibits both inhibitory and inductive effects on CYP enzymes, with evidence from *in vitro*, animal, and clinical studies indicating altered drug metabolism. These findings highlight the need for careful therapeutic monitoring when Ginkgo is co-administered with medications metabolized by CYP450 enzymes, particularly in patients on multiple drugs or with a narrow therapeutic index.

### 4.4 Danshen (*Salvia miltiorrhiza*)

Danshen, also known as Chinese sage (*Salvia miltiorrhiza* Bunge), is a perennial plant of the Lamiaceae family native to Central and South China [115]. Its roots are highly valued in traditional Chinese medicine. Chemical analysis has identified diterpenoids and polyphenolic compounds as key constituents [116]. Processing methods influence sample characteristics more than geographical origin, with salvianolic acids, trijuganone B, cryptotanshinone, and 15, 16-dihydrotanshinone serving as quality markers [116].

Danshen has been used to treat cerebrovascular and cardiovascular disorders. Tanshinone IIA, a major diterpene, demonstrates cardioprotective, adaptogenic, neuroprotective, anti-inflammatory, and antitumor effects [117, 118]. Co-administration of Danshen tablets with atorvastatin significantly reduces Cmax, AUC, and half-life, suggesting accelerated metabolism *via* CYP3A4 induction, likely mediated by salvianolic acid B [119]. Similar interactions may occur with other CYP3A4-metabolized statins such as simvastatin and lovastatin. Danshen tablets (100 mg/kg for 7 days) also decreased amlodipine pharmacokinetic parameters, linked to elevated CYP3A4 activity [120]. Tanshinone IIA has been shown *in vitro* to upregulate CYP3A4 expression *via* PXR activation in a dose- and time-dependent manner [121].

Danshen constituents exert diverse effects on CYP isoenzymes. Salvianolic acid B induces CYP3A4 and CYP2C9, enhancing metabolism of drugs like losartan, whereas tanshinone IIA may inhibit the same enzymes, slowing clearance [105]. Studies on Danshen components-including dihydrotanshinone I, tanshinone I, tanshinone IIA, cryptotanshinone, danshensu, and salvianolic acids A-C-show that lipophilic compounds, particularly dihydrotanshinone I, inhibit CYP3A and CYP2J enzymes, influencing rivaroxaban metabolism [122].

Tanshinones act as potent CYP1A2 inhibitors and moderate CYP2C9 inhibitors, while water-soluble constituents generally show minimal CYP inhibition, except for tanshinol and salvianolic acid B. Tanshinol inhibits CYP1A2, CYP2C8, CYP2C9, and CYP2C19, and protocatechuic aldehyde reduces CYP3A4 activity [123, 124]. Lipophilic tanshinones show more pronounced inhibition on CYP1A2, CYP2C9, and CYP2E1 [125].

Clinical evidence indicates that Danshen modulates clopidogrel pharmacokinetics. In a study of 20 healthy volunteers, co-administration of Danshen reduced clopidogrel Cmax and AUC while increasing clearance and volume of distribution, consistent with CYP induction [126]. Another study suggested enhanced clopidogrel efficacy due to CYP inhibition, particularly CYP1A2 [127]. Co-administration of Fufang Danshen dripping pill with clopidogrel altered pharmacokinetics of both agents, linked to CYP2C11 and CYP3A1 induction and carboxylesterase 1 inhibition [128].

Further studies assessed 15 Danshen-derived compounds for CYP2C8 and CYP2J2 inhibition. Salvianolic acid A acted as a competitive CYP2C8 inhibitor and mixed-type CYP2J2 inhibitor, salvianolic acid C showed moderate inhibition, and dihydrotanshinone I exhibited noncompetitive CYP2J2 inhibition and mechanism-based CYP2C8 inhibition [129]. Hydrophilic constituents like salvianolic acid B showed mild inhibition, while lipophilic tanshinones were more potent. Salvianolate (salvianolic acid B, rosmarinic acid, lithospermic acid) inhibited CYP3A4 with limited effects on other CYPs [125].

In HepG2 cells, Danshen and its constituents induced CYP enzymes. Tanshinone IIA and cryptotanshinone activated PXR and, to a lesser degree, the constitutive androstane receptor and glucocorticoid receptor. They also induced CYP1A1/2 *via* the aryl hydrocarbon receptor, with up to a 14-fold increase. Danshen additionally improves liver function in non-alcoholic fatty liver disease by decreasing CYP1A2, CYP2B6, and CYP1B1 expression [130, 131]. Tanshinone IIA protects against lithocholic acid-induced cholestasis *via* PXR and CYP3A11/13 upregulation [121], although other studies report worsened cholestasis through CYP3A11 inhibition [132].

Danshen water extract protects against paracetamol-induced hepatotoxicity by inhibiting CYP2E1 [133], and compound Danshen dripping pill reduces azilsartan metabolism *via* downregulation of CYP2B1, CYP2C6, and CYP2C11 [134]. Human studies show Danshen inhibits caffeine metabolism *via* CYP1A2 [135]. Danhong injection strongly inhibits CYP2A6, moderately affects several other CYPs, and upregulates CYP3A4 mRNA [136].

Overall, Danshen exhibits complex, dose-dependent effects on CYP450 activity, with both inhibitory and inductive mechanisms depending on constituent profile. Caution is advised when co-administering Danshen with CYP-metabolized medications. Other Lamiaceae species, *i.e.* *Scutellaria baicalensis*, have been reported to regulate the aryl hydrocarbon receptor-CYP1A axis [137].

### 4.5 Garlic (*Allium sativum*)

Garlic (*Allium sativum* L.) is a perennial herbaceous plant from the Alliaceae family [138]. It possesses a wide range of therapeutic properties, including antiviral, antihelminthic, choleretic, and hypolipidaemic effects [138, 139]. Garlic also has potential cancer-preventive activity and is reported to enhance immune function [140]. Its anticancer properties are largely attributed to organosulfur compounds such as diallyl sulfides, S-allyl mercaptocysteine, and allicin [141].

Garlic is often discussed alongside other spices such as ginger, turmeric, black cumin, saffron, black pepper, and chilli pepper, all of which show promise for cancer prevention and treatment. These bioactive components exert their effects mainly by inducing apoptosis, inhibiting tumour proliferation and invasion, and enhancing tumour sensitivity to chemotherapy and radiotherapy [141]. Garlic’s biologically active sulfur- containing compounds may compete with other substrates for CYP450-mediated metabolism or, in some cases, inactivate CYP enzymes, thereby influencing drug bioavailability [142]. Consequently, widespread use of garlic as a dietary supplement has prompted substantial interest in its potential to interact with medications *via* CYP450 modulation.

Allicin is formed when fresh garlic is crushed, converting the precursor alliin to allicin through alliinase. Owing to its instability, allicin rapidly decomposes into derivatives such as diallyl sulfide (DAS), diallyl disulfide (DADS), and diallyl trisulfide (DATS) [143]. Different garlic preparations-fresh extract, garlic oil, or aged garlic extract-vary significantly in chemical composition, resulting in different pharmacological and metabolic profiles [144]. Aged garlic extract primarily contains stable, water-soluble compounds (*e.g.* S-allylcysteine), whereas fresh or oil-based preparations are rich in volatile sulfur compounds. Water-soluble constituents found in aged garlic extract show minimal effects on CYP isoforms *in vitro*, whereas allicin-rich preparations display more pronounced CYP modulation [52, 145].

Numerous *in vitro* studies indicate that garlic components can directly affect CYP450 activity. Extracts from raw garlic inhibit CYP2C9, CYP2C19, CYP3A4, and related isoforms, while exerting little effect on CYP2D6 [146]. Pure allicin exhibits potent inhibition of CYP1A2 in recombinant systems [147]. Volatile sulfur compounds show stronger inhibitory activity than water-soluble components; for example, allicin has an IC_50_ of approximately 5 µM against CYP2C9, demonstrating higher potency than its activity on CYP3A4 [146].

Animal studies demonstrate that garlic’s influence on CYP activity is dose-, time-, and isoenzyme-dependent. Eight days of fresh garlic juice in mice increased hepatic CYP1A2 and CYP2E1 expression, indicating induction [148]. Conversely, in rats, a single high dose (200 mg/kg) of DAS reduced CYP2E1 protein levels by nearly 45%, with sustained effects upon repeated dosing. DADS produced a more moderate (~25%) reduction, while S-allyl cysteine showed no effect [149]. Prolonged exposure to garlic oil components induced CYP1A1, CYP1A2, CYP2B1, and CYP3A1 several- fold, again demonstrating that garlic constituents may both inhibit and induce different CYP isoenzymes.

Human studies generally show weak or negligible effects of garlic supplements on CYP450 activity. A two-week study in 14 healthy subjects found no changes in the metabolism of dextromethorphan (CYP2D6 marker) or alprazolam (CYP3A4 marker) following garlic extract intake [150]. A 2017 meta-analysis of nine trials evaluating herb-warfarin interactions (CYP2C9 substrate) reported no significant influence of garlic-fresh, powdered, or aged-on warfarin pharmacokinetics or INR, regardless of CYP2C9 genotype [151]. Similar findings were reported for caffeine (CYP1A2), midazolam (CYP3A), and debrisoquine (CYP2D6) metabolism in controlled trials, with the exception of a minor effect on CYP2E1 in a 28-day, placebo-controlled study of concentrated garlic oil [150].

Nevertheless, specific clinically relevant interactions have been documented. Early case reports suggested up to a 50% reduction in saquinavir plasma concentrations in patients using garlic supplements, initially attributed to CYP3A4 induction. However, a 21- day controlled study showed no changes in hepatic or intestinal CYP3A4 expression, but a ~30% increase in intestinal P-glycoprotein, indicating enhanced efflux as the likely cause of decreased saquinavir absorption [152]. Ritonavir exposure showed interindividual variability-some patients experienced decreased AUC (~17%), while others experienced increases-suggesting weak induction or inhibition of CYP3A4 and P-glycoprotein depending on genetic or compositional factors in garlic preparations [150].

Collectively, data indicate that garlic’s effects on CYP450 enzymes depend on preparation type, active compound content, dosage, duration of use, and individual genetic variability. Fresh garlic and oil-based extracts rich in allicin and allyl sulfides exhibit greater potential for CYP modulation, whereas aged garlic extract-rich in S-allylcysteine and devoid of allicin-exerts minimal inhibitory effects. Clinical trials comparing aged garlic extract and garlic powder consistently show no significant influence on warfarin kinetics or coagulation parameters [151, 153]. The low oral bioavailability and rapid metabolism of allicin likely account for the limited clinical translation of the substantial *in vitro* CYP inhibition observed [154].

Overall, *Allium sativum* can modulate CYP450 enzyme activity *in vitro* and in animal models, with allicin and related organosulfur compounds displaying inhibitory effects on CYP2C9, CYP2E1, CYP3A4, and CYP1A2, and both induction and inhibition seen across various experimental settings. In contrast, controlled human studies generally show minimal effects on major CYP isoforms, supporting garlic’s relatively safe profile with respect to drug interactions. Nonetheless, modest influences-particularly on CYP2E1 and intestinal P-glycoprotein-warrant caution when high- dose garlic supplements are used concomitantly with drugs possessing a narrow therapeutic index.

### 4.6 Ginger (*Zingiber officinale*)

Ginger (*Zingiber officinale* Roscoe) is a perennial herb with a fleshy, branched rhizome belonging to the Zingiberaceae family [155]. Its native range extends from India to southern Central China. The rhizome is widely used as a spice owing to its distinctive pungent flavour and has long been incorporated into traditional Chinese medicine. Ginger contains several biologically active constituents, including gingerols, shogaols and zingerone, which are primarily responsible for its pharmacological effects. To date, more than 160 compounds have been isolated from ginger, including volatile oils, gingerol analogues, phenylalkanoids, diarylheptanoids, sulfonates and monoterpenoid glycosides [156]. Fresh ginger converts into dried ginger upon dehydration, resulting in a distinct phytochemical profile and differing therapeutic properties [157]. This highlights ginger’s potential as a versatile nutraceutical. Ginger exhibits a broad range of biological activities, including gastrointestinal protection, antimicrobial effects, immune modulation, anti-obesity actions, and anti-cancer properties [156].

Shaukat *et al.* [158] reported that 6-gingerol is the most abundant and pharmacologically important constituent of ginger. During storage or thermal processing, parts of 6-gingerol convert into shogaols, zingerone and paradols, yet gingerols remain predominant in the fresh rhizome. Consequently, the assertion that gingerol is the principal bioactive constituent is supported by contemporary evidence [159]. Ginger’s biological activity is largely mediated by its major phenolic compounds-gingerol, shogaols, paradols and zingerone. These compounds contribute to ginger’s antioxidant effects by enhancing endogenous antioxidant enzymes and glutathione levels while reducing free radical formation [160]. Gingerol also exerts pronounced anti-inflammatory effects by inhibiting nitric oxide, prostaglandin E_2_ and TNF-α production, and by suppressing NF-κB activity, thereby reducing pro-inflammatory cytokines [161]. Moreover, gingerol exhibits antimicrobial activity, demonstrating bactericidal properties against various pathogens [162]. Thus, gingerol is widely recognised as the primary mediator of ginger’s antioxidant, anti-inflammatory, antimicrobial and other beneficial effects.

Recent evidence indicates that phytochemicals from *Zingiber officinale*-including gingerols, shogaols and whole-plant extracts-may modulate the cytochrome P450 (CYP450) system through both direct enzyme inhibition and activation of nuclear receptors regulating CYP expression [163]. Qiu *et al.* performed molecular modelling of 12 major ginger constituents with human CYP1A2, CYP2C9, CYP2C19, CYP2D6 and CYP3A4, predicting substantial inhibition, particularly towards CYP2C9 and CYP3A4 [163]. Experimental findings by Husain *et al.* (2023) supported these predictions: a standardised ginger extract strongly inhibited CYP3A4, CYP2C9, CYP1A2 and CYP2B6, as well as the transporters P-gp and BCRP *in vitro* [164]. Certain individual compounds (*e.g.* 6-gingerol and 6-shogaol) were found to activate AhR and PXR, potentially inducing the expression of specific CYP enzymes [165]. Overall, ginger’s bioactive constituents can inhibit key CYP450 isoenzymes (CYP1A2, CYP2C9, CYP2C19 and CYP3A4) and, in some cases, induce them indirectly, suggesting the potential for ginger- drug interactions when used concomitantly with conventional medicines.

Clinical evidence regarding ginger’s influence on CYP450 enzymes in humans is less consistent. Multiple controlled studies have reported no significant alterations in the metabolism of CYP1A2-, CYP3A4- or CYP2C9-substrate drugs. For example, investigations using warfarin found no meaningful changes in pharmacokinetics following ginger supplementation, implying negligible impact on CYP2C9 [165]. Although *in vitro* studies suggest that gingerols and shogaols inhibit CYP1A2-raising the possibility of increased levels of caffeine or other substrates-human trials generally show minimal effects on caffeine metabolism [166]. This discrepancy likely reflects the low oral bioavailability of gingerols, resulting in systemic concentrations insufficient to elicit the degree of inhibition observed in microsomal assays.

Ginger’s effects on CYP450 enzymes vary considerably depending on the form of preparation, dosage and individual metabolic differences. Fresh ginger and its extracts contain higher levels of gingerols and shogaols and may exert more pronounced effects on CYP450 activity [167]. In contrast, aged extracts, which are richer in water-soluble compounds, tend to demonstrate weaker effects [168]. Mukkavilli *et al.* [167] found that ginger biophenols-particularly 6-, 8- and 10-gingerol-inhibit CYP1A2 and CYP2C8 in human liver microsomes. However, whole ginger extracts exerted substantially weaker inhibition than isolated compounds, suggesting that interactions among constituents within the phytocomplex may reduce overall enzyme inhibition [167]. In a Caco-2 intestinal model, none of the major ginger constituents were identified as P-gp substrates, although some compounds (*e.g.* 10-gingerol, 6-shogaol) underwent biotransformation during epithelial transport. These observations underscore ginger’s complex and preparation-specific effects on CYP450 activity and highlight the importance of evaluating whole extracts rather than isolated phytochemicals [169].

A critical analysis reveals that evidence regarding ginger’s impact on CYP450 enzymes remains inconsistent. While molecular modelling and microsomal assays predict potent inhibition-particularly of CYP3A4 and CYP2C9-clinical studies generally report minimal or no significant pharmacokinetic changes. *in vitro* work demonstrates that 6-gingerol and 6-shogaol inhibit CYP1A2, CYP2C9, and CYP3A4 at micromolar concentrations [162, 163], and standardised ginger extracts inhibit CYP3A4, CYP2C9, and CYP1A2 while modulating P-gp [163]. However, controlled human trials involving substrates such as warfarin (CYP2C9) and alprazolam (CYP3A4) show no clinically meaningful effects on drug metabolism [164, 149]. Similar discrepancies are noted for CYP1A2: although *in vitro* inhibition suggests potential caffeine accumulation, human data show negligible impact on caffeine clearance [165, 166]. The divergence between *in vitro* and *in vivo* findings likely stems from limited bioavailability of gingerols and the inability to achieve inhibitory concentrations systemically. Preparation-dependent variations further complicate interpretation; fresh ginger is rich in gingerols, dried ginger in shogaols, and aged extracts in hydrophilic constituents, each with differing CYP450 activities. These factors collectively explain the heterogeneity observed across studies.

### 4.7 Milk Thistle (*Silybum marianum*)

Milk thistle (*Silybum marianum* L. Gaertn.) is an annual or biennial herbaceous plant belonging to the Asteraceae family [170]. The primary active component of the fruit extract is silymarin, a complex mixture of flavonolignans. Among these, silybin is regarded as the major and most pharmacologically active constituent [171]. Silybin is well recognised for its hepatoprotective properties, including antioxidant, anti-inflammatory, detoxifying, antifibrotic, lipid-lowering, immunomodulatory and liver-regenerating effects [171-173]. Because its key flavonolignans-particularly silybin and isosilybin-participate in metabolic and detoxification processes, their interactions with cytochrome P450 (CYP450) enzymes have been extensively investigated to evaluate potential herb-drug interactions [174, 175]. Understanding these interactions is essential for optimising clinical efficacy and ensuring safe therapeutic use.

A recent study by Faisal *et al.* (2021) demonstrated that silybin, isosilybin and related flavonolignans form stable complexes with human serum albumin and significantly inhibit several CYP450 enzymes, particularly CYP3A4 and CYP2C9. Although sulfate metabolites generally exhibited weaker inhibition, 2, 3-dehydrosilychristin-19-O-sulfate emerged as the most potent CYP3A4 inhibitor. These findings underscore the potential for clinically relevant interactions with medications such as immunosuppressants, anticoagulants and chemotherapeutic agents metabolised by CYP3A4 and CYP2C9 [176].

Milk thistle extracts have also been shown to influence the pregnane X receptor (PXR), a nuclear receptor that regulates CYP3A4 expression in response to xenobiotics. *In vitro* gene reporter assays revealed that silybin and isosilybin markedly inhibited PXR-mediated activation of CYP3A4 expression. This suggests that milk thistle can suppress CYP3A4 induction triggered by certain medications, potentially altering drug pharmacokinetics. These properties highlight its potential as a PXR antagonist, which may help prevent CYP3A4-mediated drug-drug interactions, especially in cancer therapy [177].

Further *in vitro* work using modern chromatographic techniques identified isosilibinin (IC_50_ = 1.64 ± 0.66 µg/mL) and isosilybin B (IC_50_ = 2.67 ± 1.18 µg/mL) as the most potent CYP2C8 inhibitors among milk thistle flavonolignans, suggesting possible herb-drug interactions involving reduced clearance of CYP2C8 substrates [178]. Complementary studies confirmed that CYP2C8 plays a major role in silybin metabolism, primarily generating O-demethylated derivatives. Thus, silybin-mediated inhibition of CYP2C8 may alter the metabolism of drugs depending on this pathway, emphasising the need for further research to determine its clinical relevance [179].

Beyond CYP2C8, silymarin has been shown to modulate CYP450 enzymes involved in lipid regulation. In a recent preclinical model, the combination of silymarin with metformin altered CYP4A expression, improved hepatic lipid metabolism and reduced inflammation. These findings support silymarin’s broader potential in influencing CYP-mediated pathways related to fatty liver disease and insulin resistance [180].

Milk thistle constituents also affect phase II metabolism. UDP-glucuronosyltransferases (UGTs), a family of key conjugation enzymes, are responsible for glucuronidation. Silybin A and silybin B were identified as potent inhibitors of intestinal UGTs *in vitro*, with IC_50_ values within clinically achievable ranges. These results indicate that silymarin may influence drug clearance by inhibiting both CYP3A4 and UGT enzymes, reinforcing the importance of evaluating dietary-supplement interactions in patients with hepatic impairment or polypharmacy [181, 182].

Despite substantial *in vitro* evidence of CYP inhibition, clinical studies have yielded different conclusions. A 14-day study in healthy volunteers assessing the effects of standardised milk thistle supplementation on CYP enzyme activity reported no significant alterations in the metabolism of CYP1A2-, CYP2C9-, CYP2D6- or CYP3A4/5-substrate drugs. The lack of *in vivo* effect is likely due to poor bioavailability, rapid first-pass metabolism and the limited systemic exposure of flavonolignans [183]. Nonetheless, emerging research suggests that modulation of CYP enzymes involved in carcinogen activation may contribute to milk thistle’s potential anticancer benefits. Silymarin may inhibit CYP-dependent pathways responsible for converting pro-carcinogens into active toxins, although these mechanisms require confirmation in human studies [184].

Collectively, these findings highlight the need to consider possible interactions between milk thistle constituents and CYP enzymes, particularly when co-administered with medications metabolised by CYP3A4, CYP2C9 or CYP2C8. Healthcare professionals should carefully assess the risk of herb-drug interactions to ensure safe therapeutic use. Further well-designed clinical trials are required to clarify the pharmacokinetic implications of milk thistle supplementation and to optimise its application in clinical practice.

### 4.8 Black Cohosh (*Cimicifuga racemosa*)

Black cohosh, scientifically known as *Actaea racemosa* L. (formerly *Cimicifuga racemosa* (L.) Nutt.), is a perennial herbaceous plant belonging to the Ranunculaceae family. It is native to North America [185]. Traditionally, black cohosh has been used to treat gynaecological disorders, particularly menopausal and postmenopausal symptoms, due to its reported oestrogen- like activity [186]. Recent studies have also suggested its potential anticancer properties, particularly against breast, ovarian and cervical cancers [187]. However, black cohosh is classified as a poisonous medicinal plant, and the United States Pharmacopoeia includes warnings regarding its potential hepatotoxicity [188, 189]. Its rhizome contains several classes of bioactive compounds, including triterpenoids (primarily cycloartane derivatives such as actein, cimigenol and cimicifugoside), quinolizidine alkaloids and the isoflavonoid formononetin.

Black cohosh has been widely studied for its effects on hormonal balance, particularly its selective oestrogen receptor-modulating activity. Unlike classical phytoestrogens that directly mimic oestrogen, black cohosh constituents act through modulation of oestrogen receptors in the central nervous system, providing a possible non-hormonal alternative for alleviating menopausal symptoms [190, 191].

*In vitro* studies have demonstrated that black cohosh can inhibit several cytochrome P450 enzymes involved in drug metabolism, most notably CYP2D6 and CYP3A4. One study reported significant inhibition of CYP2D6 activity by black cohosh extracts, suggesting possible interactions with medications such as fluoxetine, metoprolol and tamoxifen [192]. Similarly, Siwek *et al.* showed that triterpene glycosides-particularly 26-deoxyactein-exerted inhibitory effects on CYP3A4 (IC_50_ = 0.027 mg/mL) [193]. Additional *in vitro* research has reported inhibition of CYP1A2, CYP2C9, CYP2D6 and CYP3A4, with compounds such as fukinolic acid and cimicifugic acids contributing to this activity. However, comprehensive reviews emphasise that despite these *in vitro* findings, there is no conclusive *in vivo* evidence demonstrating clinically significant CYP inhibition, indicating a potential but unconfirmed risk of herb-drug interactions [194].

Pang *et al.* [195] examined the effects of black cohosh on pregnane X receptor (PXR)-mediated CYP expression. In wild-type mice, black cohosh extract activated PXR and increased expression of Cyp3a11, the murine analogue of human CYP3A4. However, *in vitro* assays indicated that black cohosh does not activate human PXR, suggesting species-specific differences and implying that the risk of CYP3A4-mediated herb-drug interactions *via* PXR activation in humans is limited [195].

Additional *in vitro* work investigating commercial black cohosh formulations demonstrated that the extract did not significantly affect CYP1A1 or CYP2C9, but inhibited CYP2C19 (IC_50_ = 0.37 μg/mL), indicating a potential interaction with drugs metabolised by this isoenzyme [196]. Other recent studies confirm that black cohosh may inhibit CYP2D6, CYP2C8, CYP2C19 and CYP3A4, although findings remain inconsistent, likely due to differences in extraction techniques, phytochemical standardisation and assay conditions [77].

Black cohosh has also been investigated for its effects on phase II drug-metabolising enzymes, particularly UDP-glucuronosyltransferases (UGTs). *in vitro* studies using human liver microsomes showed concentration-dependent, moderate inhibition of UGT1A4, UGT1A6 and UGT1A9 [197]. However, the study did not assess effects on UGT2B7, a key enzyme involved in the metabolism of drugs such as morphine, irinotecan and several anticonvulsants. Thus, its full influence on UGT-mediated metabolism remains unclear and requires further investigation.

Despite consistent *in vitro* evidence of CYP and UGT inhibition, clinical studies have not demonstrated significant alterations in CYP enzyme activity following black cohosh supplementation in humans. Factors such as poor oral bioavailability, extensive first-pass metabolism and rapid systemic clearance may explain the discrepancy between *in vitro* and *in vivo* findings [52].

Overall, current evidence suggests that black cohosh has the potential to inhibit several CYP450 and UGT enzymes, but clinically relevant herb-drug interactions remain unproven. Nonetheless, given the variability in commercial preparations and the potential for hepatotoxicity, caution is warranted when black cohosh is co-administered with medications metabolised by CYP2D6, CYP3A4, CYP2C19, or UGT isoforms. Further well-designed clinical studies are needed to determine the clinical significance of these interactions.

### 4.9 Ginseng (*Panax ginseng*)

Ginseng (*Panax ginseng* C.A. Mey) is a perennial herbaceous plant. *Panax ginseng* belongs to the Araliaceae family and is commonly found in the far eastern regions of the Eurasian continent [198]. It is considered one of the most important traditional medicines globally. Recent genomic studies have enabled the systematic identification of genes involved in the biosynthesis of ginseng’s main bioactive compounds, known as ginsenosides, which are tetracyclic glycosylated triterpenoids [198, 199]. Additionally, ginseng roots contain valuable polysaccharides, polyphenols, volatile terpenoids, and polyacetylenes [199]. The primary bioactivities of *Panax ginseng* include adaptogenic, nootropic, anti-ageing, immunoregulatory, anti-diabetic, and anti-cancer effects [200]. As a result, ginseng root preparations are frequently prescribed as tonics and adaptogens for conditions such as fatigue, reduced cognitive performance, and diabetes.

It is essential to examine how *Panax ginseng* interacts with CYP enzymes, as these enzymes are key regulators of drug metabolism, and their alteration may result in potential herb-drug interactions. A deeper understanding of these interactions is crucial for predicting possible metabolic changes that could impact the efficacy and safety of co-administered medications.

A study involving healthy participants assessed the impact of *Panax ginseng* on CYP3A4 activity using midazolam as a probe substrate. After 28 days of ginseng administration (500 mg twice daily), results indicated a significant reduction in the area under the concentration-time curve of midazolam by 34%, its half-life by 29%, and its maximum concentration by 26%. These findings suggest that *Panax ginseng* may increase hepatic and possibly gastrointestinal CYP3A4 activity [201]. Additionally, the study demonstrated that careful clinical monitoring is recommended for patients taking warfarin or medications metabolized by CYP3A or P-glycoprotein, especially those with narrow therapeutic indices [202].

In contrast, recent studies show that *Panax notoginseng* has a low potential to induce CYP3A activity, minimising the risk of herb-drug interactions. In one study using primary rat and human hepatocytes (*in vitro*) and human microbiota-associated rats (*in vivo*), repeated oral administration of *Panax notoginseng* root extract did not stimulate CYP induction in hepatocytes or elicit hepatic Cyp3a1/Cyp3a2 (rats) or human CYP3A4/CYP3A5 expression. The study also demonstrated that increased microbial deglycosylation, rather than enzyme induction, accounted for elevated systemic levels of oxidised metabolites. These findings support the safe co-administration of *Panax notoginseng* root extract in cardiovascular treatments, particularly in polypharmacy settings where CYP3A-mediated interactions are a concern [203].

Additionally, another study showed that red ginseng extract has minimal potential for clinically significant herb-drug interactions involving CYP enzymes and organic anion-transporting polypeptide 1B1 (OATP1B1), as the pharmacokinetic profiles of probe substrates remained largely unchanged following 15 days of use. *In vitro* studies further confirmed that ginseng had no significant effects on the activity of major enzymes and transporters responsible for drug metabolism. These results suggest that red ginseng is well tolerated and unlikely to cause major CYP- or OATP-mediated drug interactions, supporting its safe co-administration with other medications [204].

In this context, a comprehensive review analysed interactions between medicinal plants and drug-metabolising systems involving *Panax notoginseng* and its bioactive components. The review highlighted both synergistic and adverse effects when combined with conventional drugs. Pharmacodynamic interactions indicate that *Panax notoginseng* enhances the therapeutic effects of anticancer, antiplatelet, and antimicrobial agents, whereas pharmacokinetic interactions show its ability to influence drug metabolism and transport, mainly through CYP enzymes and P-glycoprotein. These findings underscore the need for caution when co-administering *Panax notoginseng* with drugs that have a narrow therapeutic window [205].

Recently, it was shown that *Panax ginseng* panaxytriol induces CYP3A4 expression by enhancing pregnane X receptor recruitment to steroid receptor coactivator 1 and acetyltransferase P300, facilitating its binding to the ER-6 and DR-3 response elements. Furthermore, this regulation is modulated by constitutive androstane receptor expression, as the effect of the pregnane X receptor is further enhanced when the constitutive androstane receptor is silenced. These results highlight the complex interactions between the constitutive androstane receptor and pregnane X receptor in drug metabolism and offer new insights into the regulatory mechanisms of CYP3A4 activation [206]. Such knowledge may improve understanding of herb-drug interactions and inform the clinical use of compounds derived from *Panax ginseng*.

Despite some evidence suggesting that ginseng can modulate CYP enzyme activity, clinical studies have often shown minimal or no significant interactions. For example, a clinical study assessing fermented red ginseng on CYP enzymes and P-glycoprotein activity in healthy volunteers reported no significant interactions with CYP probe substrates following two weeks of administration. However, red ginseng significantly inhibited P-glycoprotein activity, suggesting a potential for increased systemic exposure to drugs that depend on P-glycoprotein for transport [207].

This result was supported by a recent well-controlled clinical trial in which healthy participants received *Panax ginseng* (100 mg twice daily for 14-28 days). Pharmacokinetic assessments using probe substrates showed no clinically relevant alterations in the activity of CYP1A2, CYP2C9, CYP2D6, CYP3A4, or P-glycoprotein, as measured by the metabolism of midazolam, caffeine, debrisoquine, and fexofenadine. However, *in vitro* studies have demonstrated that specific ginseng metabolites, especially those that are deglycosylated, can upregulate the mRNA expression of CYP1A1, CYP1A2, and CYP3A4 in HepG2 cells and human liver microsomes. These findings suggest a mechanistic potential for enzyme modulation, but this has not translated into meaningful clinical effects *in vivo*, even after repeated dosing [208].

Another study examined how *Panax ginseng* affected the pharmacokinetics of lopinavir and ritonavir, HIV protease inhibitors, in healthy participants. The study concluded that two weeks of administration did not alter the steady-state pharmacokinetics of either drug, suggesting that a clinically significant interaction is unlikely [209].

Overall, *Panax ginseng* exhibits a wide range of effects on CYP enzymes, depending on factors such as dosage, duration, preparation type, and individual metabolic differences. While certain studies suggest that ginseng or its constituents can affect CYP enzyme activity, clinical evidence frequently shows little to no significant interactions. Nevertheless, due to the variability in findings and potential for individual differences, it is advisable to monitor patients closely when *Panax ginseng* is co-administered with drugs that rely heavily on CYP metabolism, particularly those with narrow safety margins.

### 4.10 Liquorice (*Glycyrrhiza glabra*)

Liquorice (*Glycyrrhiza glabra* L.) belongs to the Fabaceae family and is primarily found in the temperate regions of Eurasia [210]. Research has demonstrated that this plant contains a variety of phytocompounds, primarily triterpenoids and flavonoids [211]. The main bioactive constituents include 18β-glycyrrhetinic acid, glabrin A and B, glycyrrhizin, and isoflavones [210]. These compounds exhibit a range of pharmacological activities, including anti-inflammatory, antiviral, antimicrobial, phytoestrogenic, detoxifying, anticarcinogenic, and antidiabetic effects [140, 211].

It is essential to investigate the interactions between liquorice constituents and CYP enzymes, as these enzymes play a central role in drug metabolism. Modulation of CYP activity could lead to clinically relevant herb-drug interactions, and understanding these effects can help predict pharmacokinetic changes that might enhance or reduce drug efficacy [212].

*In vitro* studies have shown that liquorice modulates CYP450 enzyme expression and activity, exhibiting inhibitory effects on CYP2D6, CYP3A4, and CYP1A2. CYP2D6 and CYP3A4 were shown to be dose-dependently regulated [10]. *Glycyrrhiza glabra* has also been reported to modulate CYP3A4 activity through the PXR pathway. Both *in vitro* and *in vivo* models confirmed this effect. *In vitro*, human PXR was dose-dependently activated by glabridin, while glycyrrhizin and glycyrrhetinic acid did not directly activate PXR. However, in PXR- and CYP3A4-expressing mice, oral administration of glycyrrhetinic acid (100 mg/kg/day for 4 days) significantly increased hepatic CYP3A4 mRNA, protein expression, and enzymatic activity, suggesting PXR-dependent induction *in vivo*. These results indicate that certain *Glycyrrhiza glabra* components, particularly glycyrrhetinic acid, may enhance CYP3A4-mediated drug metabolism, highlighting the potential for herb-drug interactions at high or concentrated doses, especially in polyherbal preparations [213].

A separate *in vitro* analysis identified licoisoflavone B as a key inhibitor, displaying reversible competitive inhibition of CYP2C8 and mixed-type inhibition of CYP2C9, with Ki values of 7.0 ± 0.7 μM and 1.2 ± 0.2 μM, respectively. It also moderately inhibited CYP2B6 *via* both reversible and irreversible mechanisms [214]. Liquorice polysaccharides, the primary water-soluble constituents of *Glycyrrhiza glabra*, were shown to non-competitively inhibit CYP46A1 (Kᵢ = 0.7003 mg/mL) *in vitro* and reduce 24S-hydroxycholesterol levels *in vivo*. They also decreased CYP46A1, HMG-CoA reductase, and NMDA receptor subunit protein levels without affecting mRNA expression, suggesting post-transcriptional regulation [214].

Further studies, combining *in vitro* mechanisms with pharmacokinetic assessments, demonstrated that Glycyrrhiza species and their active phytochemicals significantly modulate CYP3A4 and CYP1A2 activity, potentially impacting the metabolism and clearance of antiviral drugs such as rilpivirine and dolutegravir. Among various Glycyrrhiza species, Glycyrrhiza echinata exhibited the greatest induction of CYP3A4, whereas G. uralensis and G. inflata strongly induced CYP1A2, highlighting species-specific differences in their effects on drug-metabolizing enzymes. Compounds such as glabridin, licoisoflavone A, glyasperin C, and glycycoumarin played key roles in activating PXR and AhR, thereby modulating CYP enzyme activity [215].

A phase 1 clinical study using a standardized *Glycyrrhiza glabra* extract, depleted of glycyrrhizic acid, showed no clinically relevant pharmacokinetic interactions with CYP3A4/5, CYP2C9, CYP2D6, or CYP1A2. Minor changes in time to Cmax and elimination half-life were observed for caffeine and tolbutamide, but these were not sufficient to impact drug metabolism or therapeutic outcomes [206]. Extending this research, an *in vivo* study assessing the combined effect of Glycyrrhizae Radix and Euphorbiae Pekinensis Radix on CYP2C9 activity using tolbutamide as a probe substrate found that this combination significantly increased CYP2C9 activity, reducing tolbutamide plasma levels. This indicates that concurrent use of these herbs could reduce the efficacy of drugs metabolized by CYP2C9 [215].

While *in vitro* studies suggest that *Glycyrrhiza glabra* and its constituents can inhibit various CYP enzymes, clinical studies have generally not demonstrated significant pharmacokinetic interactions *in vivo*. Nevertheless, the potential for herb-drug interactions cannot be entirely dismissed, particularly when *Glycyrrhiza glabra* is consumed in combination with other herbs or medications metabolized by CYP enzymes. Healthcare providers should remain aware of these potential interactions to prevent adverse effects, and further studies are needed to fully clarify their clinical impact.

The summarized data regarding the effect of the ten medicinal plants described above on CYP450 enzymes are presented in Table **2**.

## 5 OTHER PLANTS AND PHYTOCONSTITUENTS THAT CAN TARGET CYP450

The use of medicinal cannabis has surged remarkably in recent years, driven by widespread legalization worldwide [216]. Cannabis-based products are increasingly used to manage various health conditions, often concurrently with prescribed medications. This polypharmacy heightens the risk of clinically significant herb-drug interactions, a major concern for healthcare providers, as serious adverse events can result, particularly when interactions involve CYP-mediated drug metabolism.

Research has shown that cannabidiol (CBD) and delta-9-tetrahydrocannabinol (THC), the two primary bioactive phytoconstituents of *Cannabis sativa*, act as reversible inhibitors of several cytochrome P450 enzymes [217]. While both compounds exhibit inhibitory effects *in vitro*, only CBD demonstrates time-dependent inhibition. Bansal *et al.* [218] further confirmed that CBD, THC, and their metabolites inhibit CYP enzymes in human liver microsomes in both time-dependent and reversible manners. Static modeling predicted that significant pharmacokinetic interactions may occur with oral CBD at 700 mg when co-administered with drugs primarily metabolized by CYP3A. Similarly, inhaled (75 mg) or oral (130 mg) THC is predicted to interact with drugs metabolized predominantly by CYP2C9. Another study demonstrated that 12 cannabinoids inhibit CYP-mediated drug metabolism, particularly CYP2C9, at clinically relevant concentrations [216]. Cannabinoids and major THC metabolites were also shown to inhibit multiple CYP450 enzymes, with preliminary modeling indicating potential interactions with drugs extensively metabolized by CYP2C9, CYP2B6, and CYP2D6 [219].

Other phytomedicines, such as kava-kava (*Piper methysticum*) and common valerian (*Valeriana officinalis*), have been reported to inhibit intestinal metabolism of midazolam in rats [220]. Additionally, medicinal plants like goldenseal and kava-kava can modulate the activity of specific CYP isoforms, although their use is more prevalent in the “New World” than globally [77].

Cannabis interactions with anticancer medications have also been discussed, involving drug transporters, cytochromes, and glucuronyl transferases [221]. Given their anti-inflammatory and immunosuppressive properties, cannabinoids are being explored as adjuncts to checkpoint inhibitor immunotherapy [222]. Case reports evaluating potential interactions with oral anticoagulants indicate that CBD may alter the pharmacokinetics of warfarin-containing products [223].

The study conducted in Nigeria assessed the potential risk of herb-drug interactions involving five medicinal plants: *Ocimum gratissimum*, *Moringa oleifera*, *Picralima nitida*, *Vernonia amygdalina*, and *Azadirachta indica* [224]. Using *in vitro* assays, the research found that the extracts exhibited a potential for time-dependent inhibition of six cytochrome P450 enzymes when incubated at a concentration of 0.2 mg/mL.

Several natural products, herbal teas, and synthetic analogues have been screened for inhibition of CYP2B6-mediated methadone metabolism [225]. Molecular modeling revealed that dihydromethysticin and 2, 2'-dihydroxychalcone bind at the CYP2B6 active site, while gambogic acid binds allosterically. These compounds may inhibit CYP2B6 and methadone metabolism at clinically relevant concentrations.

Large-scale screening of ethanolic extracts from 123 medicinal plant species assessed potential herb- drug interactions *via* nuclear receptors, drug-metabolizing enzymes, and transporters. Thirteen plants inhibited CYP3A4, and ten inhibited CYP1A2, with IC_50_ values ranging from 1.3 to 10 μg/mL [226]. Specific extracts from Cat’s Claw, Feverfew, Red Clover, Peppermint Oil, and Siberian Eleutherococcus were evaluated, showing that Peppermint Oil, Cat’s Claw, and Siberian Eleutherococcus inhibited CYP3A4 more effectively than 0.1 μM ketoconazole in fAd-CYP3A4 cells [227].

Case reports also indicate that compounds from *Astragalus membranaceus*, *Schisandra sphenanthera*, and certain fruits, such as grapefruit and cranberry, act as definitive CYP inhibitors [7].

In general, while many herbal medicinal products and supplements demonstrate therapeutic potential, the clinical efficacy of several remains unverified. Their use is often inadequately monitored, leading to limited understanding of mechanisms, contraindications, adverse reactions, and potential interactions with conventional drugs or functional foods [228]. This underscores the importance of careful evaluation and monitoring of herbal product use, especially in patients taking medications with narrow therapeutic indices or metabolized *via* CYP enzymes.

## CONCLUSION AND FUTURE PERSPECTIVES

This review highlights the complex and intricate mechanisms through which various herbal compounds modulate CYP450 enzyme activity, leading to significant pharmacokinetic alterations that can impact drug efficacy and safety. Our analysis emphasizes that, although herbal products are often perceived as inherently safe, their diverse phytochemical profiles render them susceptible to both pharmacodynamic and pharmacokinetic interactions. The CYP450 enzymes, central to drug metabolism, are primary targets for such modulation. Both induction and inhibition of these enzymes by specific phytochemicals can result in clinically relevant outcomes, ranging from therapeutic failure due to accelerated drug clearance to adverse effects stemming from elevated drug concentrations.

A well-documented example is St. John’s wort, which exhibits potent inductive effects on key CYP isoforms, particularly CYP3A4. The hyperforin content in St. John’s wort preparations is a critical determinant of the magnitude of these interactions, underscoring the importance of using standardized herbal extracts and considering dose-dependent effects. CYP3A4 induction by St. John’s wort can substantially reduce the systemic exposure and efficacy of numerous co-administered drugs, including oral contraceptives and certain cardiovascular medications.

In addition to St. John’s wort, other medicinal plants-including Echinacea, Ginkgo, Danshen, Ginger, Milk Thistle, Black Cohosh, Ginseng, and Liquorice-have been shown to modulate CYP450 enzymes. However, findings in this area are sometimes contradictory. For instance, Echinacea may exhibit both inductive and inhibitory effects on CYP isoforms. Such discrepancies highlight the need for rigorous, well-controlled clinical studies to accurately characterize the interaction profiles of widely used herbal medicines. Furthermore, inherent genetic variability in CYP enzyme expression among individuals adds another layer of complexity, influencing both the predictability and clinical manifestation of herb-drug interactions.

The widespread use of herbal medicines alongside conventional drugs presents a significant challenge in clinical practice. Healthcare providers must remain vigilant regarding potential herb-drug interactions, particularly those mediated by the CYP450 system. A comprehensive patient history, careful monitoring of drug safety, and awareness of herbal supplement use are essential. Future research should focus on standardized herbal products, pharmacogenetic factors, and well-designed clinical trials to establish clear, evidence-based guidelines for the safe and effective co-administration of herbal products with conventional medications.

## Figures and Tables

**Table 1:** Examples of completed clinical trials regarding herb-drug interactions (ClinicalTrials.gov).

Official Title	ClinicalTrials.gov ID	Location	Condition	Study Type	Intervention / Treatment	Subjects	References
Clinical Evaluation of the Pharmacokinetic Goldenseal-Metformin Interaction in Diabetic Patients	NCT05081583	Mary Paine, Washington State University, United States	Diabetes mellitus type 2	Interventional (Phase 1)	Drug: Midazolam Hcl 1Mg/Ml Inj Dietary Supplement: Goldenseal (*Hydrastis canadensis*) 20 subjects were administered a single dose of goldenseal (3.3 g) orally, 30 minutes before to the administration of midazolam	22 (18 Years to 65 Years (Adult, Older Adult	[14]
Herb-Opioid Interactions	NCT00027014	Seattle, Washington, United States	Pain	Interventional (Phase 4)	Drugs: St. John's Wort (*Hypericum perforatum*) and 2 narcotic (opioid) pain medications: oxycodone and fentanyl	54 (21 Years to 45 Years - аdults, all sexes	[15]
Effect of *Moringa oleifera* on Metformin Plasma Level in Type 2 Diabetes Mellitus Patients	NCT03189407	University of Ibadan, Nigeria	Type 2 diabetes mellitus	Interventional (Phase not applicable)	Dietary Supplement: *Moringa oleifera* tea patients were given fourteen sachets of pre- packed 400 g dried leaves for preparation of tea for 7 days (twice daily), and their normal doses of metformin	25 (49 Years to 77 Years, Adults)	[16]
*Moringa oleifera* - Antiretroviral Pharmacokinetic Drug Interaction	NCT01410058	Harare, Zimbabwe	HIV infections	Observational	Dietary Supplement: *Moringa oleifera* (leaf powder, 1.85g once daily, hard gelatin capsules) Drug: Efavirenz 600mg Drug: Nevirapine 200Mg Oral Tablet	19 (18 Years and older, Adults)	[17]
Drug Interactions Between *Echinacea purpurea* and Etravirine	NCT01347658	Barcelona, Spain	HIV	Interventional (Phase not applicable)	Dietary Supplement: *Echinacea purpurea* root (500 mg every 8 h) in addition to their antiretroviral treatment	15 (18 Years to 99 Years, adults, all sexes	[18]
Drug Interactions Between *Echinacea purpurea* and Darunavir/​Ritonavir	NCT01046890	Badalona, Barcelona, Spain	HIV infections	Interventional (Phase 4)	Drug: darunavir/ritonavir 600/100 mg + root of *Echinacea purpurea* (500 mg every 6 hours)	15 (18 Years and older)	[19]
Resveratrol and Midazolam Metabolism	NCT01173640	Seattle, Washington, United States	Healthy	Interventional (phase nоt applicable)	Drug: Midazolam Dietary Supplement: resveratrol (single dose 1 g on eighth day). Dietary Supplement: resveratrol (multiple dose: 1 g oral dose for 8 days, Between Visit Days 8 and 15)	6 (18 Years to 50 Years (Adult)	[20]
The Use of Cannabidiol in Cancer Patients (CANPADIOL)	NCT05407298	Reims, France	Cancer	Observational	Prevalence of сannabidiol consumption among patients who received anticancer treatment	350 (18 Years and older	[21]
The Interaction of Herbs and Statins	NCT05072405	Chinese University of Hong Kong, Hong Kong	Drug interaction	Interventional (Phase 4)	Drug: Simvastatin 20 mg Drug: Rosuvastatin 10 mg The green tea extract and soy isoflavones were given at a dose containing epigallocatechin gallate 800 mg (once daily) or isoflavones 120 mg (once daily for 14 days)	38 (18 years to 45 years, male adults)	[22]

**Table 2:** The effect of the selected medicinal plants on CYP450.

**CYP**	**St. John's Wort**	**Echinacea**	***Ginkgo biloba***	**Danshen**	**Garlic**	**Ginger**	**Milk Thistle**	**Black Cohosh**	**Ginseng**	**Liquorice**
**CYP3A4**	**↑↑**	**↑**	**↓/?**	**±**	**↑**	**↓/?**	**±**	**?**	**±**	**↓/?**
**CYP2D6**	**0**	**0**	**0**	**↓**	**0**	**0**	**↓**	**?**	**↓**	**0**
**CYP2C9**	**↑**	**0**	**±**	**↓**	**±**	**↓/?**	**±**	**?**	**↓**	**↓**
**CYP1A2**	**0**	**↓**	**0**	**↓**	**0**	**0**	**↓**	**?**	**↓**	**0**
**CYP2C19**	**↑**	**0**	**↑**	**↓**	**±**	**↓/?**	**↓**	**?**	**±**	**↓**
**CYP2E1**	**0**	**?**	**0**	**↓**	**↓**	**0**	**0**	**?**	**0**	**0**

**Notes:** ↑↑ - strong induction (dark green), ↑ - moderate induction (light green), ↓ - inhibition (pink), ↓/? - potential inhibition or conflicting data (reddish), ± - conflicting/mixed results (yellow), 0 - no effect (grey), ? - no data (white).

## LIST OF ABBREVIATIONS

FDA: Food and Drug Administration
EMA: European Medicines Agency
PXR: Pregnane X Receptor
UGTs: UDP-glucuronosyltransferases
OATP1B1: Organic Anion-Transporting Polypeptide 1B1
